# Change in Voice Quality with Voice Therapy, Injection Laryngoplasty, and Medialization Thyroplasty 

**Published:** 2025

**Authors:** Pushkaraj A. Kulkarni, Anagha A Joshi, Gopishankar Subramaniasamy, Manish Prajapati, Renuka A Bradoo

**Affiliations:** 1 *Department of ENT, * *Lokmanya Tilak Municipal General Hospital, Sion, Mumbai,* *Maharashtra 400022, India.*

**Keywords:** Hyaluronic acid, Injection laryngoplasty, Medialization thyroplasty, Unilateral vocal fold paralysis, Voice therapy

## Abstract

**Introduction:**

In patients with unilateral vocal fold paralysis (UVFP), voice therapy (VT), injection laryngoplasty (IL), and medialization thyroplasty (MT) are reported to produce significant improvement in voice quality (VQ). However, their long-term outcomes have not been sufficiently evaluated. We assessed the improvement in VQ with VT, IL, and MT over 12 months.

**Materials and Methods:**

This prospective observational study involved 95 adult patients with UVFP, categorized into three groups: VT (n=55), IL (n=36), and MT (n=4). The VQ was assessed with voice handicap index (VHI), maximum phonation time (MPT), phonatory gap (PG), and breathy voice quality (BVQ) on Day 1, 15, 30, 90, 180, and 360.

**Results:**

In the VT group, the decrease in PG, VHI score, and BVQ score was significant from Day 15, 30, and 30, respectively, while MPT increased significantly from Day 15 (p<0.05). In the MT group, similar changes were observed; however, these changes were significant from Day 1 (p<0.05). In both the groups, the lowest PG, VHI score, and BVQ score, and the highest MPT were attained on Day 360. In the IL group, PG, VHI score, and BVQ score decreased significantly, and MPT values increased significantly at every visit (p<0.05). However, PG, VHI score, and BVQ score started increasing and MPT started decreasing from Day 90, 15, 90, and 180, respectively.

**Conclusion:**

All treatment modalities improved the VQ significantly, immediately with IL and MT, and gradually with VT. Moreover, the improvement was long-term with VT and MT, and of intermediate duration with IL.

## Introduction

Unilateral vocal fold paralysis (UVFP), a frequently observed laryngeal disorder, is a result of dysfunction of the recurrent laryngeal nerve or anywhere in the course of the vagus nerve (1). It is mainly a result of iatrogenic injuries; however, other factors, including idiopathic, neurological, neoplastic, non-surgical trauma, inflammatory, and infectious diseases, are also implicated (2). UVFP is estimated to affect 5 per 100,000 individuals per year. With rising rates of thyroid and cervical spine surgeries, the incidence of UVFP has increased drastically, estimated at 2–10% and up to 21% following thyroid and cervical spine surgeries, respectively (3). These patients are characterized by the change in voice, hoarseness, or tendency to aspirate (4). The resulting dysphonia has considerable impact on the ability to communicate effectively and affects the quality of life, resulting in social withdrawal, psychological stress, and depression (5,6).

In patients with UVFP, various treatment modalities, based on phonatory gap (PG) and requirement of patients, include conservative, minimally invasive, and surgical approaches such as voice therapy (VT, PG < 1 mm), injection laryngoplasty (IL, PG 1 – 2 mm), and medialization thyroplasty (MT, PG > 2 to 3 mm) (7). In those with mild symptoms and sufficient airway protection, wait-and-watch and VT are preferred. While, in those with a profound PG and/or aspiration, surgery is preferred (3). Currently, surgical medialization through type I thyroplasty is the procedure of choice (8). Other surgical measures include arytenoid adduction and reinnervation. However, these surgical interventions, including IL, are reported to be equally effective in complete restoration of laryngeal symmetry or paralyzed vocal fold (VF) function (9). In these patients, an ideal approach is the combination of both VT and surgery. Use of VT prior to surgical intervention provides relief from unwanted hyperfunctional compensation and decreases the extraneous muscle activity to achieve the best surgical outcome (10). A retrospective study evaluating 51 patients with post-thyroidectomy UVFP who received VT, hyaluronic acid (HA) injection, autologous fat injection (FI), or MT reported that all treatment modalities improve the voice outcomes at 3 to 6 months following treatment (11). A prospective study demonstrated that both VT and type I thyroplasty are equally effective in patients with UVFP; however, it was limited by the small sample size (n=10 in each group) and short follow-up duration (3 months) (8). Though some of the authors have studied the long-term outcomes of IL with HA (12 to 24 months) (9,12,13), VT (12 months) (1), and MT (5 years) (14), these outcomes have not been evaluated sufficiently in a single study. Thus, we evaluated the improvement in voice quality (VQ) with VT, IL, and MT in patients with UVFP, and simultaneously evaluated the changes in voice parameters over 12 months.

## Materials and Methods

This prospective observational study was performed in the Department of Otolaryngology of a tertiary care institute over a period of 4 years (January 2018 to December 2022). The study enrolled adult (≥18 years) male and female patients presenting with UVFP who were willing to be regular for follow-up visits. Clinically, UVFP was defined as an immobile VF with or without an atrophic and bowing appearance. While the patients with primary or metastatic laryngeal malignancies, posterior glottic stenosis, and those patients who could not comply with the treatment due to their comorbidities or underlying diseases were excluded. The study commenced after obtaining approval of the Institutional Ethics Committee and written informed consent of the patients. Based on the treatment received, a total of 95 patients were categorized into three groups: VT group (n=55; 57.89%), IL group (n=36; 37.89%), and MT group (n=4; 4.21%). Additionally, the patients in IL and MT groups received VT, both before and after the intervention. Pre-intervention, the findings on laryngoscopy and voice assessment were recorded. In each patient, VT was initiated as soon as the diagnosis was confirmed. The patients with idiopathic (n = 15) or iatrogenic injuries (n = 5) with recent onset UVFP (within 1 month), where only compression or stretch of nerve was observed without nerve transection, received a short course of steroids tapered over 10-14 days (15). IL was performed in patients who either had aspiration or who required immediate voice improvement. MT was performed in patients who either had a history of UVFP for more than 1 year or in whom no vocal cord movement recovery occurred even after 1 year of non-surgical management. For all the patients, a senior therapist with more than 5 years of clinical experience conducted the sessions. The patients underwent two sessions, 30 minutes each, of individualized and monitored VT per week for the first month, and subsequently one session, 30 minutes, per week from second month onwards. The patients were trained to perform further daily sessions at home. Based on the clinical judgement, VT was individualized and included push-pull exercises, head tilt method, half-swallow boom, neck relaxation exercises, vocal function exercises, digital manipulation/ manual circumlaryngeal therapy, and humming exercises.In the IL group, the procedure was performed in the office settings under local anesthesia. Prior to the HA injection, pharynx, tonsils, vallecula, and epiglottis were sprayed with 10% lidocaine solution and laryngeal gargling was performed with 2% lidocaine solution. Through a percutaneous or a transoral technique, HA (Restylane^®^, Galderma Laboratories, Texas, United States) was injected lateral to the vocal process and the middle third of the VF at the depth of the vocalis muscle. The required amount of HA ranged from 0.5 to 1.0 ml, based on the acoustic feedback and laryngoscopic visualization of PG. The procedure lasted for around 15 minutes. In the MT group, type I thyroplasty was performed under local anesthesia in combination with light intravenous sedation. At the middle level of the thyroid cartilage, a horizontal skin incision was made, the platysma was elevated, the strap muscle was retracted, and the thyroid cartilage was exposed. At 3-4 mm above the lower margin and 6-8 mm lateral to the midline of the thyroid cartilage, thyrotomy window (3-4 mm high and 5-8 mm wide) was created. A silastic block was used to medialize the paralytic VF and the depth of medialization was adjusted based on the auditory feedback by instructing the patient to phonate intra-operatively. On ascertaining the VQ, the implant was sutured, and the wound was closed in layers. The patients were discharged on the third post-operative day. Both IL and MT were performed by the same surgeon with more than 5 years of surgical experience. Post-intervention, laryngoscopy and voice assessment was done similar to pre-intervention. Assessment was performed on regular intervals on follow-up Day 1, 15, 30, 90, 180, and 360. Parameters for voice assessment included voice handicap index (VHI), maximum phonation time (MPT), PG on rigid laryngoscopy, and breathy voice quality (BVQ) in acoustic voice analysis.

The objective evaluation of PG was done with laryngoscopy, performed with a 70º rigid laryngoscope (HOPKINS, Karl Storz, Tuttlingen, Germany). Based on the PG pattern proposed by Lundy et al., PG was graded as: 0 (none), 1 (minimal), 2 (small), 3 (moderate), and 4 (severe). Where 0 suggested no visible gap, 1 suggested a minimal gap with involvement of the posterior non-membranous part of the VF, 2 suggested a small gap reaching up to 33% of the posterior membranous VF, 3 suggested a moderate gap reaching up to 67% of the posterior membranous VF, and 4 suggested a severe gap with no visible contact between the VF (16).

### Statistical analyses

The data was analyzed with SPSS (IBM, Armonk, NY, USA) version 23.0 for Windows. The categorical and continuous variables are represented as frequency (percentage) and mean (standard deviation, SD), respectively. Repeated measures ANOVA was used to assess the change in outcome parameters over the study period. A two-tailed probability value of less than 0.05 was considered statistically significant.

## Results

The patients were predominantly female (69.47%) and belonged to 51 – 60 years age (25.26%). The majority of the patients had left-sided UVFP and the most common etiology of UVFP was idiopathic (41.05%) followed by iatrogenic (15.79%), neoplastic (15.79%), stroke (9.47%), trauma (9.47%), and infective (8.42%). The mean time interval between the onset of UVFP and patient presentation was 18.77 (9.67) days (Table 1). Based on the treatment, this interval was 21.82 (6.52), 11.06 (3.17), and 46.25 (12.50) days for VT, IL, and MT, respectively.

**Table 1:** Demographic and clinical characteristics

**Characteristics**	**n**	**%**
**Sex, n (%)**		
Male	29	30.53
Female	66	69.47
Age, years, n (%)		
≤ 30	20	21.05
31 – 40	19	20.00
41 – 50	16	16.84
51 – 60	24	25.26
61 – 70	11	11.58
> 70	5	5.26
Side affected, n (%)		
Right	39	41.05
Left	56	58.95
Time interval, days, mean (SD)	18.77 (9.67)	-
Etiology, n (%)		
Idiopathic	39	41.05
Iatrogenic	15	15.79
Neoplastic	15	15.79
Stroke	9	9.47
Trauma	9	9.47
Infective	8	8.42

In the VT group, the mean VHI score increased slightly on Day 1, but did not reach a statistically significant level (p=0.643). On subsequent follow-up visits, the mean VHI score started decreasing with maximum decrease observed on Day 360 and this reduction was statistically significant on Day 15, 30, 90, 180, and 360 compared to the baseline value (all p<0.001). Likewise, the mean PG and BVQ score started decreasing from Day 15; however, the reduction was statistically significant on Day 30, 90, 180, and 360 compared to the baseline value (all p<0.001). However, the mean MPT values increased from Day 1 and reached a statistically significant level on Day 15 (p=0.005) with maximum increase observed on Day 360 compared to the baseline value (all p<0.001) (Table 2).

**Table 2:** Intra-group comparison of outcome measures over study duration in VT group (n=55)

**Outcome measures**	**Duration**						
	**Baseline**	**Day 1**	**Day 15**	**Day 30**	**Day 90**	**Day 180**	**Day 360**
Voice Handicap Index (VHI) score							
Mean (SD)	83.20 (9.76)	83.35 (9.76)	79.65 (9.72)	73.50 (10.45)	63.95 (13.20)	53.85 (13.36)	25.53 (17.43)
p	Reference	0.643	<0.001	<0.001	<0.001	<0.001	<0.001
Maximum Phonation Time (MPT, sec)							
Mean (SD)	2.70 (0.92)	2.75 (0.91)	3.05 (1.09)	4.25 (1.11)	6.45 (1.87)	8.80 (2.09)	20.35 (5.64)
p	Reference	0.330	0.005	<0.001	<0.001	<0.001	<0.001
Phonatory Gap (PG)							
Mean (SD)	2.75 (0.44)	2.70 (0.47)	2.70 (0.47)	2.30 (0.57)	1.70 (0.47)	0.95 (0.75)	0.50 (0.82)
p	Reference	0.330	0.330	0.001	<0.001	<0.001	<0.001
Breathy Voice Quality (BVQ) score							
Mean (SD)	2.90 (0.30)	2.90 (0.30)	2.85 (0.36)	2.15 (0.58)	1.55 (0.60)	0.80 (0.52)	0.30 (0.71)
p	Reference	0.330	0.330	0.001	<0.001	<0.001	<0.001

In the IL group, the mean VHI score decreased significantly at all the follow-up visits compared to the baseline value (all p<0.001). However, the mean VHI score started increasing from Day 90 and this increase persisted till Day 360. Likewise, the mean PG and BVQ score decreased significantly at all the follow-up visits compared to the baseline value (all p<0.001). 

However, the mean PG and BVQ score started increasing from Day 15 and 90, respectively and this increase persisted till the end of the study. Additionally, mean MPT value increased significantly at all the follow-up visits compared to the baseline value (all p<0.001), with maximum increase observed on Day 90 and then the values started decreasing (Table 3).

**Table 3:** Intra-group comparison of outcome measures over study duration in IL group (n=36)

**Outcome measures**	**Duration**						
	**Baseline**	**Day 1**	**Day 15**	**Day 30**	**Day 90**	**Day 180**	**Day 360**
Voice Handicap Index (VHI) score							
Mean (SD)	84.05 (16.29)	63.80 (19.65)	55.10 (17.87)	44.10 (14.95)	47.10 (13.05)	50.15 (14.11)	54.30 (15.68)
p	Reference	<0.001	<0.001	<0.001	<0.001	<0.001	<0.001
Maximum Phonation Time (MPT, sec)							
Mean (SD)	3.65 (1.72)	6.50 (2.68)	7.95 (2.78)	9.50 (2.78)	15.50 (2.82)	12.60 (2.80)	9.20 (2.75)
p	Reference	<0.001	<0.001	<0.001	<0.001	<0.001	<0.001
Phonatory Gap (PG)							
Mean (SD)	2.85 (0.51)	0.00 (0.00)	0.75 (0.71)	1.05 (0.60)	1.20 (0.69)	1.45 (0.75)	1.85 (0.82)
p	Reference	<0.001	<0.001	<0.001	<0.001	<0.001	<0.001
Breathy Voice Quality (BVQ) score							
Mean (SD)	2.85 (0.36)	2.00 (1.07)	1.95 (1.09)	1.80 (1.19)	2.55 (0.93)	2.30 (0.86)	2.35 (0.65)
p	Reference	<0.001	<0.001	<0.001	<0.001	<0.001	<0.001

In the MT group, the mean VHI score decreased from Day 1 and reached the lowest value on Day 360, and this reduction was statistically significant at all follow-up visits compared to the baseline value (all p<0.001). Likewise, the mean PG and BVQ score decreased significantly on Day 1 and reached the lowest value on Day 360 (all p<0.001). However, the mean MPT values increased from Day 1 with a maximum increase observed on Day 360, and this increase was statistically significant at all follow-up visits compared to the baseline value (all p<0.001) (Table 4).

On Day 360, full compensation was observed in 34 (61.82%), 28 (77.78%), and 4 (100%) patients that received VT, IL, and MT, respectively.

**Table 4:** Intra-group comparison of outcome measures over study duration in MT group (n=4)

**Outcome measures**	**Duration**						
	**Baseline**	**Day 1**	**Day 15**	**Day 30**	**Day 90**	**Day 180**	**Day 360**
Voice Handicap Index (VHI) score							
Mean (SD)	95.00 (1.41)	65.40 (24.04)	62.20 (14.14)	48.80 (12.02)	45.60 (8.48)	44.50 (6.36)	38.35 (10.15)
p	Reference	<0.001	<0.001	<0.001	<0.001	<0.001	<0.001
Maximum Phonation Time (MPT, sec)							
Mean (SD)	2.50 (0.70)	7.50 (3.53)	9.25 (3.89)	11.50 (5.28)	14.50 (2.82)	15.25 (3.01)	15.50 (2.94)
p	Reference	<0.001	<0.001	<0.001	<0.001	<0.001	<0.001
Phonatory Gap (PG)							
Mean (SD)	3.00 (0.80)	0.00 (0.00)	0.50 (0.70)	0.00 (0.00)	0.00 (0.00)	0.00 (0.00)	0.00 (0.00)
p	Reference	<0.001	<0.001	<0.001	<0.001	<0.001	<0.001
Breathy Voice Quality (BVQ) score							
Mean (SD)	3.00 (1.20)	1.00 (0.52)	0.50 (0.23)	0.00 (0.00)	0.00 (0.00)	0.00 (0.00)	0.00 (0.00)
p	Reference	<0.001	<0.001	<0.001	<0.001	<0.001	<0.001

## Discussion

Available literature demonstrates that timely intervention can considerably reduce the adverse impacts of UVFP of various origins and decrease the chances of deterioration in quality of life. The principal findings of the study suggested significant improvement in VQ with all the treatment modalities, this agrees with the findings of a systematic review (4). Though the improvement was immediate with both IL and MT, it was gradual with VT. Additionally, the improvement lasted longer with VT and MT, while it was of intermediate duration with IL. The study was performed in a tertiary care center and the use of improved surgical approaches along with intraoperative nerve monitoring resulted in reduced incidence of iatrogenic UFVP. Thus, in the present study, the most common UFVP etiology was idiopathic, in contrast to the available literature which suggests iatrogenic procedures as the most common etiology of UFVP (3).

Irrespective of etiology, VT is the first-line treatment, especially in those with high vocal demand and strong motivation (3). VT decreases the adverse impacts of UVFP on daily life and assist patients endure the recovery period. Though the nature of VT may defer based on the settings, the majority of these techniques intend to improve the laryngeal compensatory mechanisms from the normal side. With VT, possible benefits can be assessed after completion of one to two sessions. It has been demonstrated that 4-6 weeks is the least amount of time required to attain sustained benefits (17,18). Early initiation of VT results in superior VQ (19), with early VT leading to long-lasting and sustained improvement in VQ for 12 months. Additionally, even delayed initiation of VT has been demonstrated to produce significant and sustained benefit for 12 months (1). We observed that the improvement in VQ was gradual and steady. In all the parameters studied, the improvement was significant from Day 15 onwards, reaching a peak at Day 360. The VQ continued to improve till 12 months and thus, the improvement persisted till the end of the study. Due to its sustained effect, VT was employed either in isolation or both before and after the surgical intervention.

We performed IL in patients who either had aspiration or required immediate improvement in VQ. In our set-up, HA is mainly used for IL, primarily due to its greater tissue compatibility, easy access, and favorable biomechanical properties (20). Early administration of HA injections may result in suitable VF position with enhanced outcomes (21), thereby explaining the fact that only a small proportion of the patients in the present study required MT. The primary benefit associated with HA injections is ease of administration under local anesthesia and performance in the out-patient set-up. It can result in rapid voice recovery. However, available literature suggests that HA injections provide symptomatic relief for 9-14 months (22), demonstrating that HA is suitable as a temporary measure of UVFP. In the present study, the improvement in VQ was immediate. The VQ continued to improve significantly up to 1 – 3 months, and then this improvement was steady for a variable period. The effect of HA lasts for a short period, due to its rapid degradation (20).

Among various treatment modalities, MT is used as a permanent treatment of UVFP and with experienced surgeon leads to satisfactory outcomes. Past studies demonstrate long-term benefits with MT up to a duration of 12 months (14). In the present study, patients with history of UVFP for more than 1 year or those with no recovery of VF movement even after 1 year of non-surgical measures underwent MT. Similar to IL, the improvement in VQ was immediate with MT. However, unlike IL, the improvement in VQ persisted and was steady for a period of 12 months. The findings further suggest that VQ was superior following MT compared to other treatment modalities.

The main strength of the study included evaluation of outcome measures at various follow-up visits, thereby highlighting the significant change in particular parameters at different intervals. Additionally, the patients received either isolated VT or combination of IL or MT with VT, thus depicting the real-world scenario observed in clinical practice. However, the study had certain limitations. First, UVFP was of various origins, thereby introducing heterogeneity in the findings. Second, the duration of UVFP and presenting symptoms varied substantially among the patients. Third, the treatments were not randomized but decided by an elective decision between the patient and the surgeon.

The findings of the present study highlight the need for multidimensional assessment of VQ. We did not intend to compare different treatment modalities regarding improvement in VQ or symptoms, as these modalities as well as their indications are different. Additionally, these modalities are not mutually exclusive, and there was an overlap of the modalities i.e., VT was given to all the patients. This is because the aim of the treatment is to keep the patient symptom- free and restore the voice. Based on the findings, we strongly recommend VT with VQ as a primary endpoint, irrespective of the duration of UVFP. To optimize voice outcomes, VT must be offered to all the patients with UVFP, as early as possible.

## Conclusion

To conclude, UVFP is mostly idiopathic in nature and all the three treatment modalities provide significant improvement in VQ. The improvement in VQ was immediate with both IL and MT, but it was gradual and steady with VT. Also, VT and MT led to long-term improvement, while the improvement was of intermediate duration with IL. Overall, the findings demonstrated long-term effectiveness of VT in patients with UVFP. Though the treatment needs to be tailored for individual patients, VT should be combined with all the treatment modalities.

## Source(s) of Support

Nil.

## Presentation at a Meeting

Nil.
